# Supplementary material for: COZOID: contact zone identifier for visual analysis of protein-protein interactions
Source: BMC Bioinformatics. 2018 Apr 6;19:125. doi: 10.1186/s12859-018-2113-6 (PMC5889581; doi:10.1186/s12859-018-2113-6)
Supplement: Supplementary file 2 — Software build. Executable binary file of the software tool. (ZIP 118,784 kb) [file 12859_2018_2113_MOESM2_ESM.zip › cozoid/platform/docs/testng-6.5.1-javadoc.zip!/org/testng/FileAssert.html]

FileAssert (TestNG)


---


|  |  |  |
| --- | --- | --- |
| **Overview** \| **Package** \| **Class** \| **Tree** \| **Deprecated** \| **Index** \| **Help** | |  |
| **PREV CLASS**   **NEXT CLASS** | **FRAMES**    **NO FRAMES**     **All Classes** |  |
| SUMMARY: NESTED \| FIELD \| CONSTR \| METHOD | DETAIL: FIELD \| CONSTR \| METHOD |  |


---


## org.testng Class FileAssert

```
java.lang.Object
  org.testng.FileAssert
```

---

``` public class FileAssert extends java.lang.Object ```

Assertion tool for File centric assertions.
Conceptually this is an extension of `Assert`
Presents assertion methods with a more natural parameter order.
The order is always **actualValue**, **expectedValue** [, message].

**Since:**
:   5.6

**Version:**
:   $Revision: 650 $, $Date: 2009-01-05 03:51:54 -0800 (Mon, 05 Jan 2009) $

**Author:**
:   Paul Mendelon

---

| **Method Summary** | |
| --- | --- |
| `static void` | `assertDirectory(java.io.File tstvalue)` |
| `static void` | `assertDirectory(java.io.File tstvalue, java.lang.String message)`             Asserts that a `tstvalue` is a proper directory. |
| `static void` | `assertFile(java.io.File tstvalue)` |
| `static void` | `assertFile(java.io.File tstvalue, java.lang.String message)`             Asserts that a `tstvalue` is a proper directory. |
| `static void` | `assertLength(java.io.File tstvalue, long expected)` |
| `static void` | `assertLength(java.io.File tstvalue, long expected, java.lang.String message)`             Asserts that a `tstvalue` is a file of exactly `expected` characters or a directory of exactly `expected` entries. |
| `static void` | `assertMaxLength(java.io.File tstvalue, long expected)` |
| `static void` | `assertMaxLength(java.io.File tstvalue, long expected, java.lang.String message)`             Asserts that a `tstvalue` is a file of at most `expected` characters or a directory of at most `expected` entries. |
| `static void` | `assertMinLength(java.io.File tstvalue, long expected)` |
| `static void` | `assertMinLength(java.io.File tstvalue, long expected, java.lang.String message)`             Asserts that a `tstvalue` is a file of at least `expected` characters or a directory of at least `expected` entries. |
| `static void` | `assertReadable(java.io.File tstvalue)` |
| `static void` | `assertReadable(java.io.File tstvalue, java.lang.String message)`             Asserts that a `tstvalue` is readable. |
| `static void` | `assertReadWrite(java.io.File tstvalue)` |
| `static void` | `assertReadWrite(java.io.File tstvalue, java.lang.String message)`             Asserts that a `tstvalue` is readable and writeable. |
| `static void` | `assertWriteable(java.io.File tstvalue)` |
| `static void` | `assertWriteable(java.io.File tstvalue, java.lang.String message)`             Asserts that a `tstvalue` is writeable. |
| `static void` | `fail()`             Fails a test with no message. |
| `static void` | `fail(java.lang.String message)`             Fails a test with the given message. |
| `static void` | `fail(java.lang.String message, java.lang.Throwable realCause)`             Fails a test with the given message and wrapping the original exception. |

**Methods inherited from class java.lang.Object**

`clone, equals, finalize, getClass, hashCode, notify, notifyAll, toString, wait, wait, wait`

**Method Detail**

### assertDirectory

```
public static void assertDirectory(java.io.File tstvalue,
                                   java.lang.String message)
```

:   Asserts that a `tstvalue` is a proper directory. If it isn't,
    an AssertionError, with the given message, is thrown.

    :   **Parameters:**: `tstvalue` - the file to evaluate: `message` - the assertion error message

---


### assertDirectory

```
public static void assertDirectory(java.io.File tstvalue)
```

---


### assertFile

```
public static void assertFile(java.io.File tstvalue,
                              java.lang.String message)
```

:   Asserts that a `tstvalue` is a proper directory. If it isn't,
    an AssertionError, with the given message, is thrown.

    :   **Parameters:**: `tstvalue` - the file to evaluate: `message` - the assertion error message

---


### assertFile

```
public static void assertFile(java.io.File tstvalue)
```

:   **See Also:**: `assertFile(File, String)`

---


### assertLength

```
public static void assertLength(java.io.File tstvalue,
                                long expected,
                                java.lang.String message)
```

:   Asserts that a `tstvalue` is a file of exactly `expected` characters
    or a directory of exactly `expected` entries. If it isn't,
    an AssertionError, with the given message, is thrown.

    :   **Parameters:**: `tstvalue` - the file to evaluate: `message` - the assertion error message

---


### assertLength

```
public static void assertLength(java.io.File tstvalue,
                                long expected)
```

:   **See Also:**: `assertLength(File, long, String)`

---


### assertMinLength

```
public static void assertMinLength(java.io.File tstvalue,
                                   long expected,
                                   java.lang.String message)
```

:   Asserts that a `tstvalue` is a file of at least `expected` characters
    or a directory of at least `expected` entries. If it isn't,
    an AssertionError, with the given message, is thrown.

    :   **Parameters:**: `tstvalue` - the file to evaluate: `message` - the assertion error message

---


### assertMinLength

```
public static void assertMinLength(java.io.File tstvalue,
                                   long expected)
```

:   **See Also:**: `assertMinLength(File, long, String)`

---


### assertMaxLength

```
public static void assertMaxLength(java.io.File tstvalue,
                                   long expected,
                                   java.lang.String message)
```

:   Asserts that a `tstvalue` is a file of at most `expected` characters
    or a directory of at most `expected` entries. If it isn't,
    an AssertionError, with the given message, is thrown.

    :   **Parameters:**: `tstvalue` - the file to evaluate: `message` - the assertion error message

---


### assertMaxLength

```
public static void assertMaxLength(java.io.File tstvalue,
                                   long expected)
```

:   **See Also:**: `assertMaxLength(File, long, String)`

---


### assertReadable

```
public static void assertReadable(java.io.File tstvalue,
                                  java.lang.String message)
```

:   Asserts that a `tstvalue` is readable. If it isn't,
    an AssertionError, with the given message, is thrown.

    :   **Parameters:**: `tstvalue` - the file to evaluate: `message` - the assertion error message

---


### assertReadable

```
public static void assertReadable(java.io.File tstvalue)
```

:   **See Also:**: `assertReadable(File, String)`

---


### assertWriteable

```
public static void assertWriteable(java.io.File tstvalue,
                                   java.lang.String message)
```

:   Asserts that a `tstvalue` is writeable. If it isn't,
    an AssertionError, with the given message, is thrown.

    :   **Parameters:**: `tstvalue` - the file to evaluate: `message` - the assertion error message

---


### assertWriteable

```
public static void assertWriteable(java.io.File tstvalue)
```

:   **See Also:**: `assertWriteable(File, String)`

---


### assertReadWrite

```
public static void assertReadWrite(java.io.File tstvalue,
                                   java.lang.String message)
```

:   Asserts that a `tstvalue` is readable and writeable. If it isn't,
    an AssertionError, with the given message, is thrown.

    :   **Parameters:**: `tstvalue` - the file to evaluate: `message` - the assertion error message

---


### assertReadWrite

```
public static void assertReadWrite(java.io.File tstvalue)
```

:   **See Also:**: `assertReadWrite(File, String)`

---


### fail

```
public static void fail(java.lang.String message,
                        java.lang.Throwable realCause)
```

:   Fails a test with the given message and wrapping the original exception.

    :   **Parameters:**: `message` - the assertion error message: `realCause` - the original exception

---


### fail

```
public static void fail(java.lang.String message)
```

:   Fails a test with the given message.

    :   **Parameters:**: `message` - the assertion error message

---


### fail

```
public static void fail()
```

:   Fails a test with no message.


---


|  |  |  |
| --- | --- | --- |
| **Overview** \| **Package** \| **Class** \| **Tree** \| **Deprecated** \| **Index** \| **Help** | |  |
| **PREV CLASS**   **NEXT CLASS** | **FRAMES**    **NO FRAMES**     **All Classes** |  |
| SUMMARY: NESTED \| FIELD \| CONSTR \| METHOD | DETAIL: FIELD \| CONSTR \| METHOD |  |


---
